# Buffalo Medical and Surgical Association

**Published:** 1885-04

**Authors:** Frank H. Potter

**Affiliations:** Secretary


### Buffalo Medical and Surgical Association.

*Meeting of February 3, 1885.*

The President, Dr. F. W. Bartlett, in the Chair; Dr. Frank H. Potter, Secretary.

*Practice by Midwives*—The committee appointed to consider the advisability of regulating the practice of midwives for the city of Buffalo reported in favor of such regulation. This committee (consisting of Dr. Hartwig as chairman, Dr. H. R. Hopkins, Dr. William Ring, Dr. P. W. Van Peyma, and Dr. John H. Pryor) recommended that, in view of the serious evils connected with the practice of ignorant midwives, the legislature be requested to pass a bill compelling the examination and licensing of midwives in and for the county of Erie. A rough draft of a bill was then presented providing for a Board of Examiners, and for the examination and registration of all persons other than physicians now practicing, or who shall hereafter be desirous of practicing, midwifery in this county. It was the opinion of the meeting that the recommendations should be acted upon, and the bill was then referred back to the same committee, with instructions to arrange the matter in proper shape to be presented to the legislature, to obtain legal advice if necessary, and to report at a subsequent meeting.

In the discussion following the committee's report, Dr. Moody protested against any attempt to regulate the practice of midwives in the way proposed. The fault was in the medical colleges in graduating incompetent men and allowing them to begin practice without practical experience in obstetrics.

Dr. Granger could see no relation between the failings of the medical colleges and the proposal to regulate the practice of midwives. It is a question to be decided by itself. A certain amount of skill and knowledge was required in the practice of midwifery, and it was proper for the profession to see that each person, before beginning this practice, possessed at least a minimum amount of experience. This bill does not abolish the midwife; it merely regulates their practice.

Drs. Putnam, Grosvenor, Hartwig, Coakley, Van Peyma, Mann, Strong and Bartlett also spoke in favor of some regulation of the practice of midwives, and the bill was then referred to the committee, with the instructions already mentioned.

*Persons Discharged from Asylums as not Insane*—Dr. William D. Granger read a paper upon this subject, the interest of which turned upon the relation of the profession to patients before their admission to an asylum. Before a patient could be admitted it was necessary that two phys cians should be convinced that insanity existed, that they should make a sworn statement to this effect, and that this statement should be approved by a judge of a court of records. Every certificate must contain the words, "*Is Insane*," or the commitment would not be legal. Upon the patient and the certificate being presented at an asylum, the superintendent was obliged to receive him. There was no discretionary power in the matter. All the authorities agreed upon this point, and it was a just provision, both to the court, to the physicians, and to the patient. The patient having been admitted, it became the duty of the asylum physicians to make a full diagnosis and refer him to the proper ward for treatment. If full evidence of insanity was not present at the time of the first examination, it was the duty of the superintendent to keep the patient under close observation until a definite conclusion could be formed. This was necessary because the patient might have "concealed delusions, hallucinations, and illusions," and the first examination not suffice for their discovery. Then, again, the case might be of a paroxysmal character, and the patient be admitted during the intermission, when further observation would, of course, be necessary. Under this strict plan, no person was retained in an asylum who was not insane. In Utica, during the past forty years, 268 patients had been discharged as not insane, an average of 6.5 per annum. In Buffalo, during four years, 23 had been discharged, an average of 5.8 per annum. During the past year, eleven have been discharged from Utica ; from Buffalo, ten ; and it would be observed that this was considerably above the average. Now, the discharge of these persons did not reflect any discredit upon the physicians who had made the certificates of insanity. The patients were generally found to be suffering from one of the following diseases : delirium tremens, dipsomania, meningitis, cerebro-spinal meningitis, hysterical mania, hysterical epilepsy, or active delirium from other causes which were obscure ; and it might be questioned whether an asylum was not a proper place for them. Again, in the cases of persons coming from the poorer classes, the physicians had often to decide between the jail, where they found the patient confined, and an asylum, and wisely preferred the latter. This was certainly humane, and deserved commendation.

The term insanity, as interpreted by the law, might easily be made to include the diseases above mentioned. The last enactment upon this subject read as follows : " The terms 'lunacy,' 'lunatic' and 'insane,' as used in this act, shall include every species of insanity, and extend to every deranged person and to all of unsound mind, other than idiots." From this it appeared that the discharge of patients, suffering from the above-mentioned diseases, as not insane, did not reflect upon the skill of the physicians who had made the certificates.

The author then proceeded to consider how much truth there was in the sensational accounts of the attempted incarceration of sane persons in the asylums. He had recently noticed in a reputable newspaper of Cleveland, Ohio, a statement to the effect that half of the persons detained as patients in the lunatic asylums of that State were sane, and unlawfully kept in those institutions. Charges against the asylums, in this respect, had been made by newspapers in this State. It was hardly necessary to say to the members of this association that they were entirely unwarranted. In order to make it possible to incarcerate a sane person in an asylum, it was necessary to assume that the friends of the patients were designing, the physician who signed the certificate corrupt, the judge willing to be bribed, and the superintendent unscrupulous and dishonest. The conspiracy, as it was frequently called, involved too many absurd suppositions. As a matter of fact, an attempt of this kind was exceedingly rare, and was always discovered. During thirty years but three attempts had been made at Utica, and they had failed in the end. No attempt of this kind had been made at the Buffalo asylum.

The author also wished to compliment the profession on the character of the certificates presented at the asylum. They showed an amount of care and professional skill that was highly commendable. Only in a few instances was there anything wrong or objectionable found in them.

Dr. Van Peyma merely wished to ask where the line was that divided sanity from insanity. He had heard it said that all people were insane, that it was merely a question of degree, which, if true, would often make a decision very difficult.

Dr. Chas. A. King considered it impracticable to decide upon the sanity, or insanity, of a person, upon one examination. Two, or even more, examinations should be required before a physician is allowed to write a certificate. By this means, many mistakes could be avoided, as, for instance, mistaking hysteria and dipsomania for insanity. He did not think it possible to incarcerate a sane person in an asylum under the present laws. This idea is a myth, intended to frighten weak-minded people, and it is kept alive by evil-disposed persons for their own ends.

Dr. Putnam, after complimenting the author of the paper, wished he had extended his remarks so as to include eccentrics and epileptics. Were persons properly placed in these two classes often sent to the asylum as insane? He believed the mistakes made were generally to be attributed to the difference in definitions. Each teacher defined insanity to fit his own theories, and the general practitioner was often at a loss as to what to believe. For instance, in France, attempted suicide was a proof of insanity, but in this country it was not so, the person making the attempt being punished as a criminal.

Dr. Coakley had observed the ordinary practice in committing the insane to the asylums, in a distant State, and was much pleased with the care required by the authorities in this State. He thought that in some respects improvement could take place, but, on the whole, the practice was quite satisfactory.

Dr. Mann said the tables were sometimes turned, the asylum authorities making the mistakes, not the physicians who write the certificates. He reported a case which the asylum physician discharged as sane, the patient being able to cover up his delusions, and which caused a great deal of trouble. All parties concerned in the care of the insane should be particularly careful and painstaking in their observations, to avoid all possibility of blundering.

Dr. Hartwig reported a case of mania lasting three days. In this case a mistake would have been made had the patient been committed to an asylum.

Dr. Bartlett thought the danger was in too much delay in the commitment. But it was for the interest of the patient that he should be placed, early in the disease, in a place to be properly treated.

In closing the discussion, and replying to Dr. Van Peyma, who asked where the line was drawn that divided sanity from insanity, Dr. Granger said that insanity was a *disease.* It was as absurd to say that every one was more or less insane as it would be to say that everybody had typhoid fever, or phthisis, or any other disease. Its diagnosis was to be determined by a knowledge of the physiology of the nervous system, and of the derangements produced in it by disease. That it was sometimes difficult to recognize this disease in its early stages or in its obscure forms was no more to be wondered at than the difficulty in recognizing incipient phthisis or any other disease that was subtle in its approach. It was often difficult to decide as to the sanity or insanity of a person, but the physician must be convinced of the presence of disease before he wrote the certificate.

Dr. Frank W. Hinkel was then proposed for membership in the association, and he was duly elected.

Diseases reported as prevailing since the last meeting were scarlet fever, diphtheria, dysentery, measles, and cerebro-spinal meningitis.

Dr. Hartwig presented an ovarian cyst, with the history of its removal.

The society then adjourned.

FRANK H. POTTER, *Secretary.*

